# A framework for developing community‐focused medical physics outreach programs

**DOI:** 10.1002/acm2.13413

**Published:** 2021-09-10

**Authors:** Andrew P. Santoso, Sydney Jupitz, Christie Lin

**Affiliations:** ^1^ Department of Medical Physics University of Wisconsin–Madison Madison Wisconsin USA; ^2^ Department of Radiation Oncology University of Colorado Anschutz Medical Campus Aurora Colorado USA; ^3^ OnLume Inc. Madison Wisconsin USA

**Keywords:** diversity, education, equity, inclusion, outreach

## Abstract

**Purpose:**

In STEM education and careers, underrepresented minorities (URMs) experience higher attrition than non‐URM counterparts. Informal educational experiences, such as outreach, have been identified to increase URM awareness and enrollment in STEM. The objectives of this work were to (1) elucidate the current state of racial and ethnic diversity in medical physics and (2) provide a community‐focused framework for building effective outreach programs geared toward K‐12 URM students and their families.

**Methods:**

Self‐reported racial and ethnic identity data from the American Association of Physicists in Medicine (AAPM) members were obtained to identify the percentage of URM members. Outreach programming was developed for home or away events. Home events occurred at the University of Wisconsin–Madison Department of Medical Physics; away events occurred at public community institutions that served URM and economically disadvantaged populations. Demonstrations, hands‐on activities, and presentations covered radiation detection, radiotherapy, medical imaging, and medical physics career paths. High school students were asked about their awareness of medical physics prior to outreach events. Likert‐scale surveys evaluated student level of agreement (1 = *Strongly disagree* to 5 = *Strongly agree*) that home events increased their career interests in medicine and physics and interest in pursuing STEM coursework.

**Results:**

Average percentage of AAPM URM members was 10.7% from 2014 to 2020. From 2016 to 2020, 42 outreach events occurred near or within the Madison metro area. Over 1900 individuals participated in outreach events, with 50 participants on average per event. The majority of home event participants indicated their interest in medical careers increased (65.4%) and were inspired to pursue more STEM courses (73.1%) after the program.

**Conclusions:**

Our medical physics outreach program demonstrates a means of increasing awareness and interest around medical physics, particularly for underrepresented individuals. This article addresses gaps in the literature for how to create and implement effective, community‐focused medical physics outreach programs.

## 1 INTRODUCTION

Given the ever‐changing landscape of health care, the American Association of Physicists in Medicine (AAPM)’s Medical Physics 3.0 initiative seeks to “define and advocate a model of sustainable excellence in medical physics” and identifies diversity as a component of strategic goals. Additionally, AAPM's most recent diversity statement (PP 30‐B) affirms racial and ethnic diversity as a strength of workforce and efforts to increase diversity in medical physics through active outreach and mentorship are desirable.^2^ This initiative lends itself toward identifying “*specific*” efforts that promote diversity, equity, and inclusion (DEI) as an actionable means of carrying out this initiative.

These initiatives and challenges are not unique to the field of medical physics. Discussions on the importance of DEI have included the enhanced ability for teams to solve complex problems more efficiently, and address how the untapped economic resource prevailing from underrepresented minorities (URMs), inclusive of women, racial and ethnic minorities, and persons with disabilities, may address the projected supply and demand issues within STEM workforces.^3^, ^4^, ^5^, ^6^, ^7^, ^8^ Lightfoote et al. identified URMs not only lack representation in both diagnostic radiology and radiation oncology physician workforces, but also rank near the bottom in terms of representation compared to the United States and other graduate medical education specialities.^9^, ^10^ Covington et al. have also identified gender disparities exist in AAPM leadership positions.^11^ Although Van Zyl et al. identified medical physics focused outreach programs playing a key role in promoting and increasing diversity in the field, few examples exist in the literature.^8^

Community engagement with science through outreach has been shown to be an effective way to increase awareness of various opportunities within science and build relationships with the larger nonscientific community.^12^, ^13^ Beyond STEM literacy, science outreach promotes discussion and mutual understanding between scientists and participants and increases community trust in both science and medicine. Through our work shared in this article, we aim to promote an individual's enthusiasm of STEM and ability to perceive themselves as scientists (i.e., STEM identity), especially for URMs who face active barriers, such as stereotype threat and systematic marginalization, when entering STEM education and careers.^14^, ^15^, ^16^, ^17^

Although opportunities such as AAPM's Diversity Recruitment through Education and Mentoring summer program have been designed to support URM undergraduate STEM students, specific efforts to increase awareness of and interest in medical physics for a broader student population as documented in the literature are minimal. Fagerstrom et al. demonstrated active‐learning outreach lessons geared toward middle school students that can easily be implemented in community settings (e.g., science fairs).^18^ This work showed increased interest in STEM in the majority of participants (75%). Informal educational experiences such as these have been identified as pivotal toward forming one's STEM identity, help maintain URM persistence in STEM, and are ultimately attributable to obtaining a STEM career; all of these appear robust to known barriers such as socioeconomic status.^19^, ^20^, ^21^, ^22^

The purpose of this article is to (1) elucidate the current state of racial and ethnic diversity in medical physics through demographic data from professional physics societies and (2) provide a community‐focused framework for building an effective outreach program geared toward K‐12 URM students and their families. Our overall goal was to increase awareness of and interest in medical physics for traditionally underrepresented K‐12 students and build relationships with the larger community. This work may serve as a guide to other medical physicists who hope to directly engage with their communities about the richness of our field and applicability to everyone's lives.

## 2 MATERIALS AND METHODS

To provide a surrogate measure of URMs in medical physics, self‐reported racial and ethnic demographic data were obtained from the AAPM membership database from 2014 to 2020 courtesy of AAPM HQ. As a means of characterizing entry into medical physics, the percentage of physics bachelor degrees awarded to URMs was obtained from publicly available data from the American Physical Society (APS).^23^ Linear regression was performed on both demographic datasets to quantify the percent change of URM membership per year. According to the APS, URMs were defined as individuals identifying as Black or African American, Hispanic or Latinx, Native Hawaiian or Other Pacific Islander, and American Indian or Alaska Native.

**FIGURE 1:**
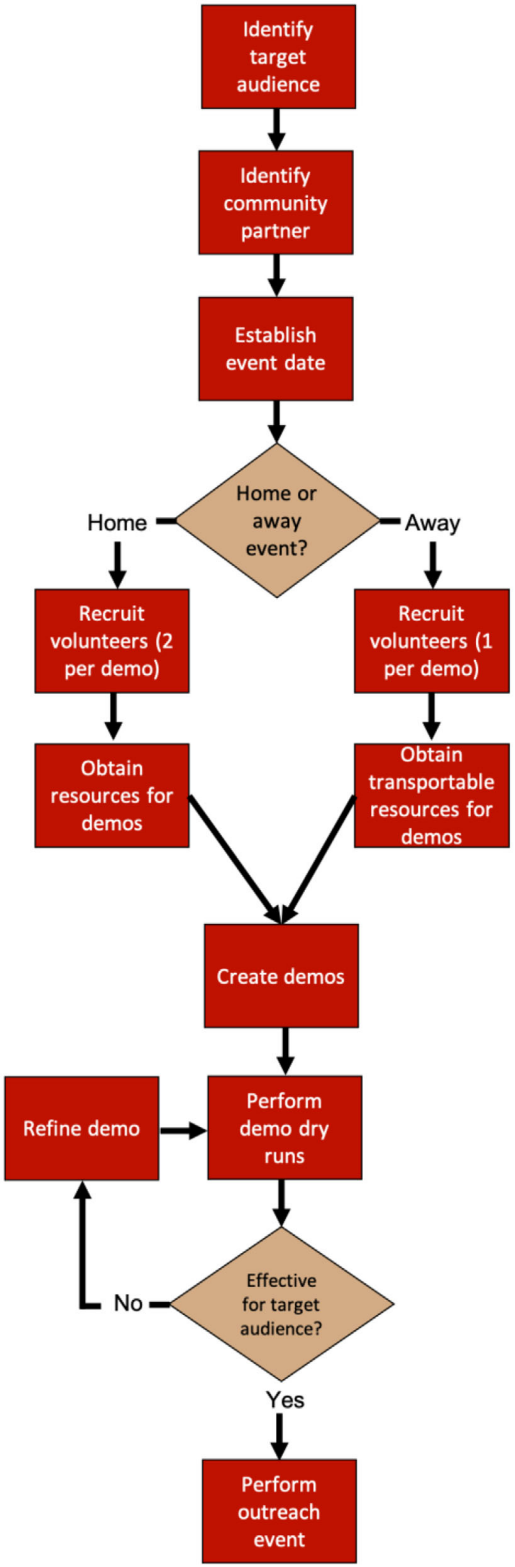
A generalized workflow for creating an outreach event designed by the UW–Madison Department of Medical Physics outreach committee. Flow branches upon deciding to hold an event at a home (e.g., clinic) or away at a community institution. In general, home events provided greater depth and required greater resources when compared to away events

With the support of our department, the University of Wisconsin–Madison (UW–Madison) Department of Medical Physics, a graduate student‐led Medical Physics Outreach Committee was formed with the expressed mission of “increas[ing] the visibility of medical physics by teaching community members about the role of physics in medicine as well as the educational and career opportunities in physics‐based careers. [The] focus is directed to the next generation of curious thinkers, especially underrepresented groups in medical physics, by engaging them in conversation and hands‐on medical physics activities.” The Outreach Committee was thus charged with developing activities, choosing engagement opportunities, and organizing outreach events. A generalized workflow of performing an outreach event is provided in Figure 1. Demonstrations, hands‐on activities, and didactic talks were created based on the type of event and target audience. Outreach events were classified as home and away events. Home events took place on‐site in our department, whereas away events occurred outside of the department and were typically hosted at off‐site locations within the local community. Away events were identified through pre‐existing outreach and community programs. Off‐site locations included public community centers and public schools, with the majority within the Madison, WI Metropolitan Area. Organizations that served URM and economically disadvantaged populations, as defined per the Wisconsin Department of Public Instruction, were prioritized for away events.^24^ Community attendees at away events spanned across all ages, ranging from kindergarten to adults. For event locations that encompassed an audience outside of K‐12 students (e.g., community centers with parents/guardians present), the “target audience” age range was identified prior to outreach events to scale demo content appropriately and ensure sufficient preparatory time for volunteer training and setting up demos. Surveys were administered for all home events (described below). A binary question of “have you heard of medical physics before today's event?” was asked at away events held at public high schools to assess awareness of the field among the community.

Home events were structured to be 3.5‐h‐long field trips, accounting for about half of a school day for participating students. Home events started with a half hour introductory session including an introduction from the department chair, testimonials from current medical physics graduate student volunteers (“volunteers”) about their education and career paths, and a brief overview of the agenda of the field trip. At the conclusion of this session, folders including the agenda, notepad, and pen were distributed to each guest student and teacher participant (“participants”). Visiting participants were then divided into four equally sized, small groups (about four to six participants per group) to rotate through the demonstration stations (2 h total). Following the full rotation through four prepared demonstration stations (half hour each), all participants rejoined for an hour‐long lunch and discussion session. During this time, volunteers were encouraged to join participants for lunch. In this way, less structured discussion and time for reflection were facilitated.

The Outreach Committee partnered with the Madison Metropolitan School District's Personalized Pathways Program, a local high school improvement strategy with the goal of closing opportunity and achievement gaps,^25^ to identify high school students to invite to the field trip. High school students participating in Personalized Pathways who identified health services as their primary career interest were invited to participate. The Pathways Program coordinator at each high school acted as the community contact and partner who knew the students and their busy school schedules best, which was invaluable knowledge for scheduling and ensuring a successful trip. Prior to home events, all high school students were required to have their legal parent or guardian sign written permission slips, indicating their child was granted permission to attend, permission to be treated by a health provider in case of an emergency, and permission to be photographed during the event.

Home events incorporated as many hands‐on demos as possible at the UW–Madison Department of Medical Physics within a 2‐h window—the target audience being high school students with a foundation of basic physics, biology, and/or medicine. A demo‐heavy tour was created, given its well‐documented positive impact on persistence in STEM education for secondary students.^19^, ^26^, ^27^ Four demonstration stations were created to showcase the diversity of medical physics topics, such as radiation detection, radiation therapy, and medical imaging. All stations were scheduled for half hour time slots and participants were divided into four equally sized, small groups. Station 1 provided a tour of the UW–Madison Accredited Dosimetry Calibration Laboratory (UWADCL), where volunteers demonstrated visualization of radiation using a Cesium‐131 check source and a large volume cloud chamber (LHS‐DC, Supersaturated Environments, Madison, WI). Station 2 focused on magnetic resonance imaging (MRI). MRI demos included scanning fruit on a 3.0 T GE MR750 MRI Scanner (GE Healthcare, Waukesha, WI) to reveal the basic MRI physics principles on how images are created. Station 3 focused on ultrasound imaging. The ultrasound demo involved hands‐on scanning of a gelatin phantom, made from a low‐cost heart‐shaped gelatin mold (<$5) and an embedded grape. The goal was for participants to identify the location of an embedded grape, which mimicked a high‐contrast lesion, using a Siemens Acuson S2000 ultrasound system (Siemens Healthcare, Mountain View, CA) while learning basic ultrasound physics principles on how images were created. Station 4 focused on the applications of ionizing radiation in the treatment of cancer. This was accomplished by describing the workflow in radiation oncology through hands‐on demos using a research radiotherapy suite. The Accuray Precision^®^ treatment planning system (Accuray, Inc., Sunnyvale, CA) was used to explain contouring, treatment planning with conventional linear accelerators, and unique aspects of helical tomotherapy. A hands‐on demo performing megavoltage computed tomography (CT) scans of two phantoms was used to describe image‐guided radiotherapy. The first iteration used a gelatin phantom, made from a low‐cost brain‐shaped gelatin mold (<$5) with a ping pong ball embedded to mimic a resection cavity. The second iteration used a multipurpose chest phantom N1 “LUNGMAN” (Kyoto Kagaku Co. Ltd, Fushimi‐ku, Kyoto, Japan) with a golf ball inserted to mimic a lung lesion.

Following all demos, high school student participants were asked to complete surveys using Google Forms to evaluate previous awareness of medical physics and program effectiveness, defined by measuring the event's impact on students’ interest in medicine, physics, and STEM. Three questions were asked on a 5‐point Likert scale in their level of agreement (1 = *Strongly disagree* to 5 = *Strongly agree*) with the following statements: (1) “As a result of this field trip, my career interests in medicine increased”; (2) “As a result of this field trip, my career interests in physics increased”; and (3) “As a result of this field trip, I am inspired to pursue more STEM courses.” Additionally, high school student participants were asked to provide free‐form text feedback to the questions, “What was your favorite learning experience during the trip and why?” and “What changes would you recommend to make your visit more interesting or effective?” for continual quality improvement of the event.

Away events involved a combination of simplified, informal hands‐on demonstrations and, for high school students, presentations about career opportunities related to medical physics. The absolute number of participants, rather than unique participants, was recorded for each event. Similar to home events, demos focused on radiation detection, medical imaging, and radiation therapy. The radiation detection demo used an Eberline E‐120 Geiger–Müller counter (Eberline Instrument Corp, Santa Fe, NM) to detect low levels of radiation from the uranium glaze of red Fiestaware™ produced circa 1930 (Homer Laughlin Co., Newell, WV).^28^ Medical imaging demos included describing how X‐ray images are produced using anonymized radiographic film, showing what can be imaged with MRI using a collection of MR image cutouts from various magazines collected over time, and demonstrating an ultrasound system mirroring the ultrasound imaging station at home events. The same gelatin phantoms with an embedded grape, described above in Station 3, were imaged using a Siemens Acuson P10 handheld ultrasound system (Siemens Healthcare). Radiation therapy demos described by Fagerstrom et al., providing a treatment planning activity and discussion of immobilization devices, were also implemented.^18^ For high school students, presentations focused on defining medical physics and the types of careers that exist in the field, specifically focusing on opportunities in academic, clinical, and industry settings. For all away events, various articles of UW–Madison Medical Physics promotional merchandise—such as pens, highlighters, flashlights, and notepads—were given away to all participants, serving as a reminder of what they learned about that day.

A primary outreach lead trained all graduate student volunteers prior to each home and away event to ensure consistent demo, activity, and talk execution. For home events, an hour‐long training was scheduled for all volunteers to discuss outreach best practices and the appropriate level of information for high school students. From experience, best practice guidelines include knowing your audience, connecting content in a way that is meaningful to your audience, and limiting lecture time to promote discussion and hands‐on investigation. Volunteers were assigned to a specific demo station based on expressed interest. During the training session, volunteers assigned to the same demo station were provided time to discuss with each other the plans for the station. The volunteers of each demo station were also tasked with providing a take‐home summary page of their station for the visiting students. For most away events, very minimal training was required. New volunteers were typically trained on‐site during preparation and setup time. They were given the opportunity to first observe an experienced volunteer interact with the audience. Every event was attended by at least one primary outreach lead to ensure volunteers felt supported in engaging with the attendees and to maintain consistency between events.

## 3 RESULTS

**FIGURE 2:**
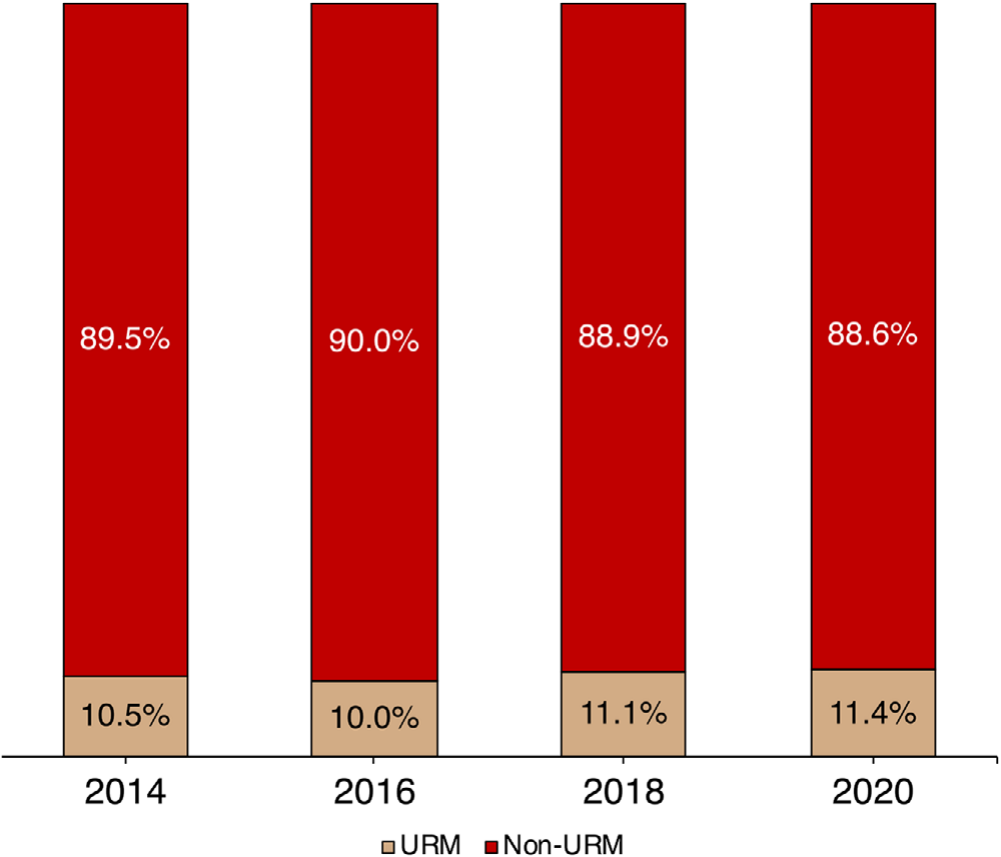
Self‐reported demographic data of AAPM membership obtained from 2014 to 2020. Underrepresented minorities (URMs) are defined as individuals identifying as Black or African American, Hispanic or Latinx, Native Hawaiian or Other Pacific Islander, and American Indian or Alaska Native. Non‐URMs constitute all other racial and ethnic identities

The average response rate of AAPM membership self‐reported racial and ethnic identity data from 2014 to 2020 was 23.6%, increasing at a rate of 2.5% per year (*R* ^2^ = 0.94). Of the available membership data, the average percentage of AAPM members identifying as a URM was 10.7%, increasing at a rate of 0.2% per year (*R* ^2^ = 0.59). The specific distribution of URM and non‐URM AAPM members as a function of year is presented by stacked bar charts in Figure 2. From 2014 to 2017, the average percentage of APS members obtaining a physics bachelors identifying as URMs was 12.1%, increasing at a rate of 0.1% per year (*R* ^2^ = 0.99).

**FIGURE 3:**
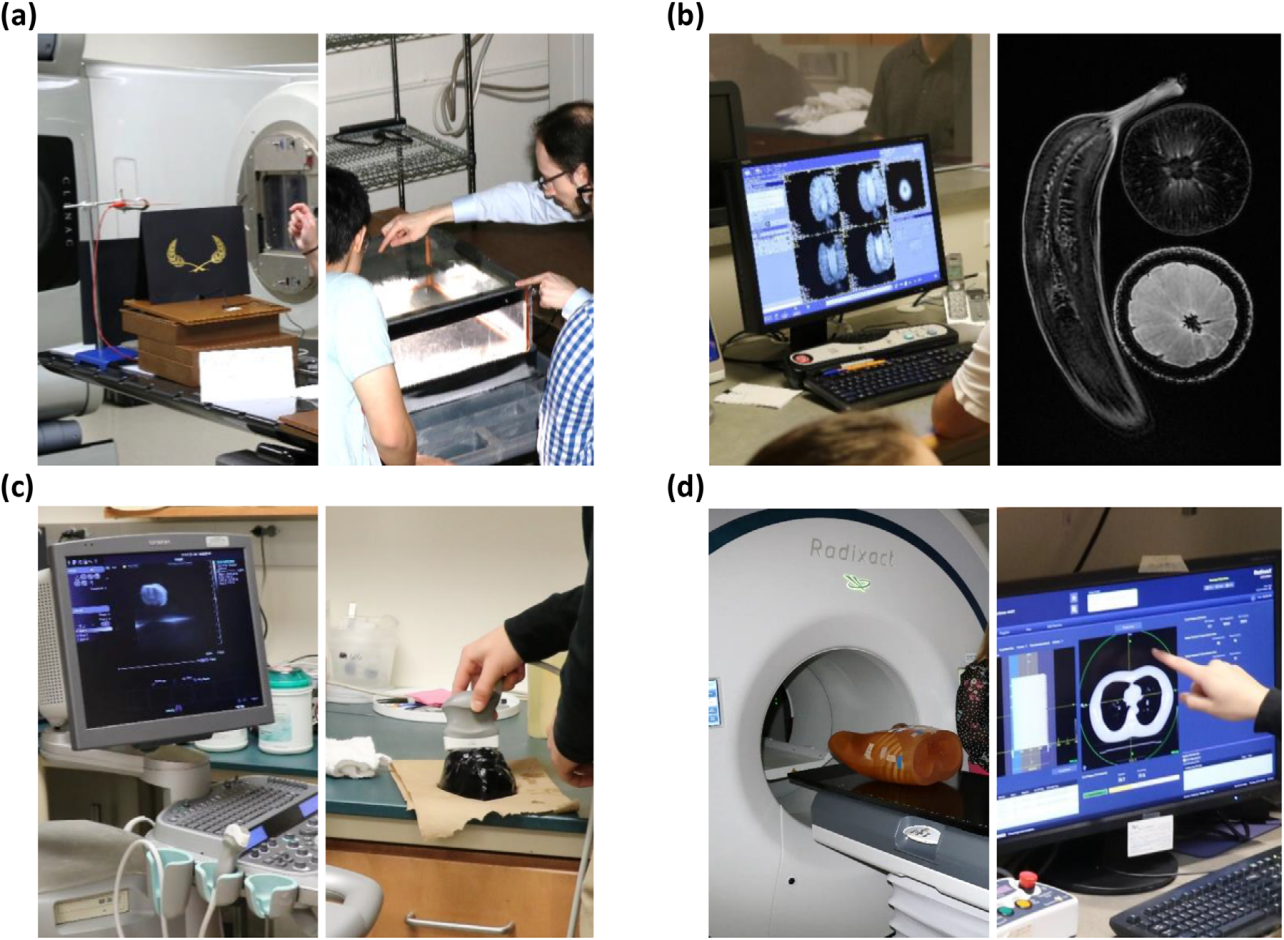
Four demonstration stations organized for high school visits to the department. Pictured are radiation detection and cloud chamber demonstrations (a), MR images of fruit (b), ultrasound imaging of a gelatin phantom with grapes inside (c), and mock patient setup and imaging in preparation for radiotherapy treatment (d)

**FIGURE 4:**
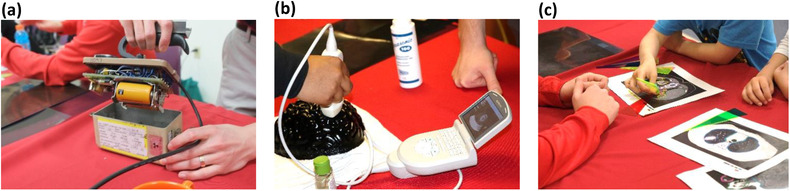
Three demonstrations taken to community events including: (a) a Geiger–Müller counter, (b) a portable ultrasound system and gelatin phantom, (c) and a treatment planning activity

Table 1 highlights event statistics related to the K‐12 medical physics outreach program, which involved over 1900 participants since its inception in 2016. Of the 42 outreach events held since program inception, 4.8% (*n* = 2) and 95.2% (*n* = 40) were home and away events, respectively. In contrast to home events, the estimated one‐on‐one interaction time between volunteers and participants was approximately 3 min. Overall, 34 different medical physics graduate students volunteered with the Outreach Committee, 19 of whom volunteered at least twice. The average number of volunteers at away events was 2, meanwhile the average number of volunteers for home events was 13. Pictures of demos from both home and away events are provided in Figures 3 and 4, respectively. Of the 125 high school student participants surveyed (response rate of 89%) about their awareness of medical physics, including participants at away events, 19.2% knew what medical physics was prior to the outreach event.

**TABLE 1:** Statistics of UW–Madison K‐12 outreach events held between October 2016 and March 2020. Note that the absolute number, rather than unique number, of participants was recorded at each away event. Time statistics for events reported in hours and minutes (h:min)

Events	
Total	42
Number of events per year, average	12
Event duration (h:min), average	2:21
Standard deviation (h:min)	1:30

Participants	
Total	1907
Number per event, average	50

Volunteers	
Total, unique MP students	34
Number of repeat volunteers	19
Number per home event, average	13
Number per away event, average	2

**FIGURE 5:**
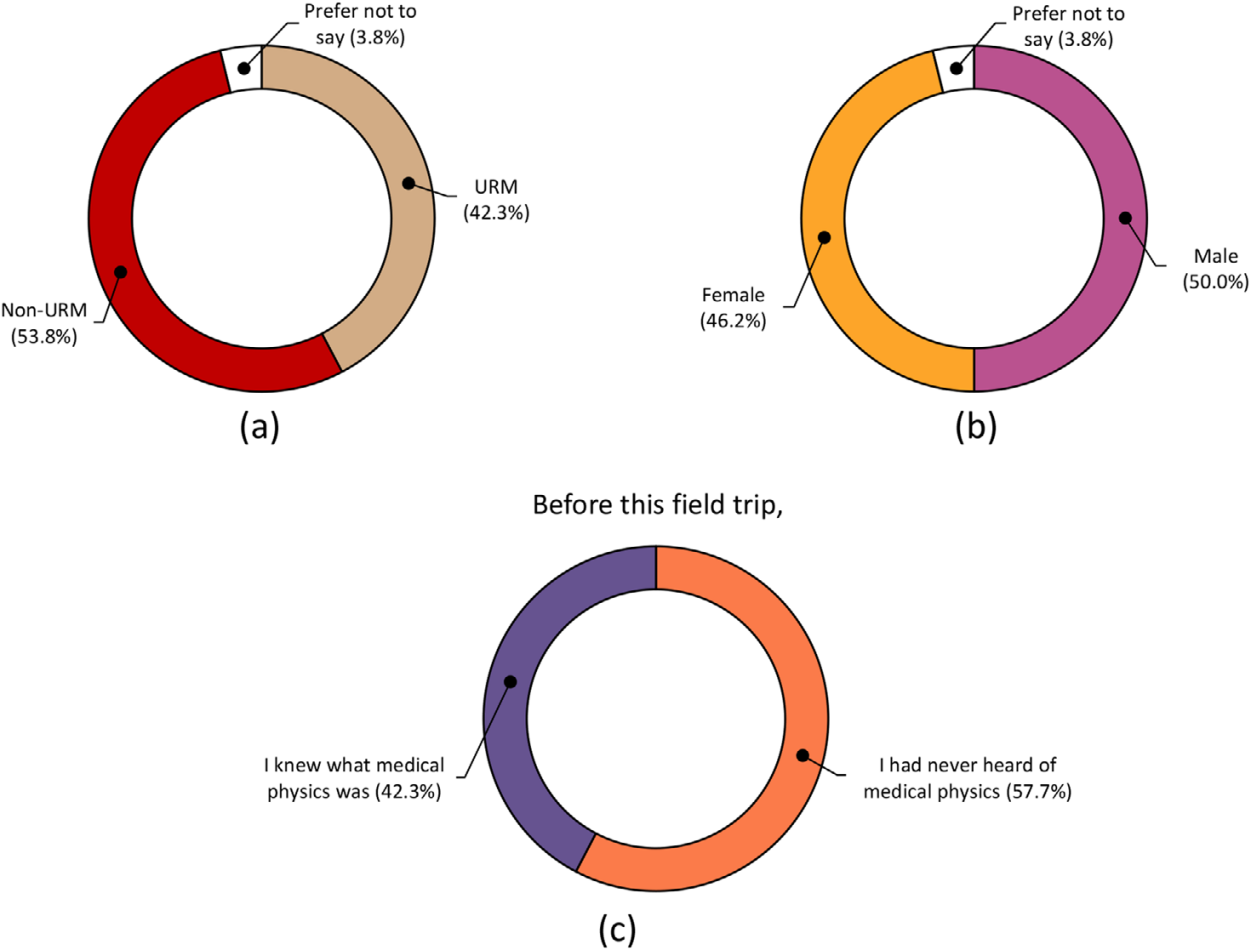
Self‐reported (a) underrepresented minority (URM) status, (b) gender identity, and (c) awareness of medical physics for high‐school students who participated in home events at the UW–Madison Department of Medical Physics. Data were collected following each home event and includes responses of 26 of 41 home event participants (63.4% response rate)

**FIGURE 6:**
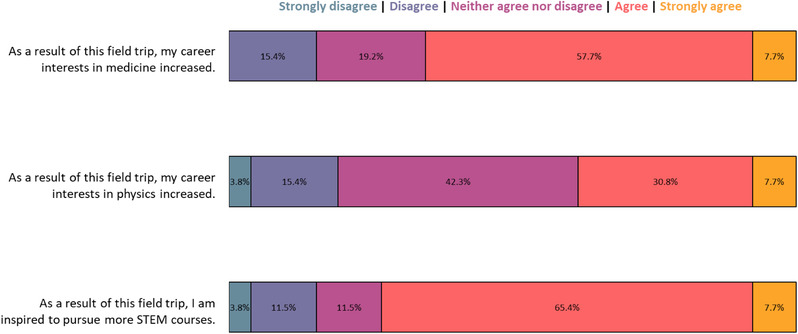
Survey results gauging career interests in medicine, physics, and STEM coursework for high school students who participated in home events at the UW–Madison Department of Medical Physics. Data were collected following each home event and includes responses of 26 of 41 home event participants (63.4% response rate)

The response rate of the 41 high school students participating in the two home events was 63.4% (26). Home event demographics and survey results from these respondents are presented in Figures 5 and 6, respectively. The majority of respondents did not know what medical physics was prior to participating in the outreach event (57.3%). Following the outreach event, the majority of respondents indicated they agreed or strongly agreed that their interest in medical or physics careers increased (65.4%) and were inspired to pursue STEM coursework (73.1%). A majority of the participants were neutral, agreed, or strongly agreed that this outreach event increased their interest in a medical physics career.

## 4 DISCUSSION

Medical physics by its nature relies on the diversity of STEM to perform tasks like noninvasive imaging to look inside the human body or treat diseases such as cancer. As indicated by a study from the Center for Health Workforce conducted between 2009 and 2010, a large sample of medical physicists in the United States (*N* = 5487) reported medical physics was a rewarding career (92%) and that they were willing to recommend the profession to others as a career (85%).^29^ However, this stated enthusiasm for medical physics falls short of, or at the very least has not manifested in, increasing recruitment of underrepresented individuals.

Despite the low response rate and limited time points from AAPM membership self‐reported racial and ethnic identity, the available data visualized in Figure 2 indicate little change in URM membership demographics. In contrast, the most recent census data indicated more than half of the United States’ population under the age of 16 identify as a racial or ethnic minority and nearly 40% of this group identify as Latinx or Hispanic and Black.^30^ The findings from the publicly available APS data indicated attrition from high school such that just over 12% of physics bachelors identify as URMs.^31^ These demographic data from both the AAPM and APS are consistent with a study on entry into STEM that demonstrated only 10% of the STEM workforce in 2010 was composed of URMs.^31^ As of 2018, the APS revised its diversity statement, which indicates that its “members” should work to increase the number of underrepresented individuals at all levels and launched a Forum on Diversity and Inclusion in 2020 to create a space focused on DEI.^32^ Other challenges of assessing URM representation via AAPM membership include insufficient demographic designations and errors in self‐report per the U.S. Office of Management and Budget. In part, AAPM created the Working Group on Equity, Diversity, and Inclusion Survey Creation and Demographic Data Collection Committee to address these problems. Although it should be acknowledged that pathways between nonphysics bachelors and medical physics still exist, these data provide an opportunity for comparison with an affiliated physics society such as the APS and reflection on the current state of medical physics racial and ethnic diversity in the United States measured by proxy through AAPM membership.^33^

As so many medical physicists know through their own experiences, our niche field remains largely unknown to the general public. Even in the high school student cohorts who participated in our outreach home events, who self‐selected toward careers in healthcare, majority (57.7%) were not aware of medical physics; furthermore, this statistic may even be inflated due to social desirability bias of knowledge in this captive audience and/or due to participants geographic proximity to our graduate program.^34^ This lack of awareness coupled with funding gaps that affect low socioeconomic status and URM students in the United States creates a complex challenge in addressing representation in STEM at large.^35^, ^36^, ^37^ A meta‐analysis of available longitudinal data conducted by Maltese and Tai indicated that merely developing an interest in STEM in eighth grade proved to be statistically significant in completion of a STEM degree.^38^ Though only 38.5% (10) of the high school outreach participants at UW–Madison home events indicated an increased career interest in medical physics, our survey indicates that the program increased interest in STEM. Although community‐based medical physics outreach events alone will not solve lack of URM representation, early and informal introductions in medical physics through outreach serve as a concrete means of addressing AAPM's Medical Physics 3.0 strategic goal pertaining to diversity, equity, and inclusion.

Opportunities for creation and growth of medical physics outreach programs are numerous. For events hosting large groups (e.g., >100 participants) such as in many away events, the short interaction times limited the means of program evaluation. In the case of away events with smaller groups, assessments similar to the work by Poole et al. were used for the purpose of program improvement and volunteer training development.^39^ Home event participants consistently provided qualitative feedback that hands‐on operation of clinical equipment under outreach volunteer supervision was a major strength of this program, as identified by students’ favorite learning experiences. On the other hand, the MRI station was identified as an improvement area, where students indicated confusion and information overload as it pertained to the physics aspects of the demo (i.e., proton precession). Although there may be interest to share current medical physics research topics with this audience, we suggest outreach content focus on fundamental medical physics building blocks to not deter from the following objectives: (1) to increase awareness of medical physics; (2) to pique student interest in our field; (3) to form one’s STEM identity early in their education; and (4) to contribute to student STEM literacy.

In terms of away events, preliminary experiences required coordination to borrow equipment (e.g., portable ultrasound scanners and Geiger–Müller counters). By merely demonstrating high‐volume interactions through photographs, the Outreach Committee was able to secure a portable ultrasound system on loan from a vendor for dedicated outreach purposes. Communicating these efforts with the rest of the department encouraged faculty and staff to share or loan equipment for demonstrations and activities. These events would not have been possible without the support of the department at large. Anecdotally, graduate student outreach volunteers frequently remarked that participating as volunteers felt personally rewarding and generated enthusiasm for volunteering at upcoming events. Identifying the opportunities for growth, subsequent needs of an outreach program, and identifying volunteers and community partners are crucial for effective execution.

In translating medical physics outreach to the broader community, it is important to recognize and identify the resources and community in which one finds themselves. Regarding resources, the majority of the equipment used in outreach programming are accessible from clinics, universities, or are of low cost. However, it is recognized that the use of clinical equipment at away events may not be feasible. In these circumstances decommissioned, nonclinical equipment used for qualitative demonstrations may be a more viable option. As it pertains to home events, the UW–Madison Department of Medical Physics represents a unique institution, with a variety of equipment dedicated for research. Consequently, challenges pertaining to patient privacy pose less of a barrier in this environment for outreach program implementation as compared to a clinic. When translating home events to clinical environments, permission slips could include language to maintain HIPAA compliance, notably the importance of patient privacy and asking students to refrain from photography during their visit. It is worth noting the increased number of average volunteers at home events is a testament to the additional work and support that home events require. As it pertains to away events, medical physicists who find themselves in rural communities or far from community centers have challenges identifying community events and/or partners similar to those indicated in this article. To overcome these obstacles, away events can occur at ubiquitous public institutions such as libraries and schools or existing STEM networks such as the National Girls Collaborative Project can be used to identify larger ongoing STEM efforts in one's own community.^40^ Though it is strongly encouraged to avoid pure didactic instruction, particularly for pre‐high school audiences, the “EDUCATORS RESOURCE GUIDE” provided by AAPM's Public Education Committee may serve as a starting point for creating outreach materials (see https://www.aapm.org/education/ERG/PUBLICED/). Two additional resources for creating effective science outreach programs are included for convenience: (1) Bevan et al. with the Center for Advancement of Informal Science Education highlight important considerations and intentions when developing a science outreach program^41^ and (2) a recent publication from McClure et al. outlines “keys to success” as takeaways from their chemistry‐focused science outreach program.^42^ Given the authors’ extensive experience, we are also happy to provide outreach materials we generated upon request. Additionally, pursuing remote learning outreach opportunities is particularly topical as of 2020, as they reduce transportation and geographical barriers and the need for in‐person programming. Though outside the scope of this article, the development of remote engagement opportunities and activities accessible online for use at home or in the classroom may serve to increase the visibility of medical physics to K‐12 students. Ultimately, individuals like you have the ability to share this rewarding field within their communities and, in part, influence the next generation of curious thinkers.

## 5 CONCLUSION

Community‐focused medical physics outreach programs for K‐12 students are effective at introducing and inspiring students to pursue careers in STEM and medicine. In alignment with AAPM's strategic goals and placing importance on DEI, outreach programs such as these also foster good community relationships and hold space for discussion and exploration, contributing to participants’ perceptions of science and STEM literacy. Although facilities at large academic institutions present unique opportunities, implementation of medical physics outreach programs from individuals or smaller institutions is quite feasible. As individuals, we play a role in shaping the field we want to see and influence the next generation of medical physicists.

## CONFLICT OF INTEREST

The authors declare no conflict of interest.

## AUTHOR CONTRIBUTION

AS and CL designed the project. AS, SJ, and CL contributed to acquisition of data. AS and SJ performed data analysis. AS drafted the manuscript. All authors read, revised, and approved the final manuscript.

## Data Availability

The data that support the findings of this study are available from the corresponding author upon reasonable request.
